# Unique Metabolomic and Lipidomic Profile in Serum From Patients With Crohn’s Disease and Ulcerative Colitis Compared With Healthy Control Individuals

**DOI:** 10.1093/ibd/izad298

**Published:** 2023-12-29

**Authors:** Hauke Christian Tews, Franziska Schmelter, Arne Kandulski, Christa Büchler, Stephan Schmid, Sophie Schlosser, Tanja Elger, Johanna Loibl, Stefanie Sommersberger, Tanja Fererberger, Stefan Gunawan, Claudia Kunst, Karsten Gülow, Dominik Bettenworth, Bandik Föh, Carlos Maaß, Philipp Solbach, Ulrich L Günther, Stefanie Derer, Jens U Marquardt, Christian Sina, Martina Müller

**Affiliations:** Gastroenterology, Hepatology, Endocrinology, Rheumatology and Infectious Diseases, Department of Internal Medicine I, University Hospital Regensburg, Regensburg, Germany; Institute of Nutritional Medicine, University Medical Center Schleswig-Holstein, Campus Lübeck, Lübeck, Germany; Gastroenterology, Hepatology, Endocrinology, Rheumatology and Infectious Diseases, Department of Internal Medicine I, University Hospital Regensburg, Regensburg, Germany; Gastroenterology, Hepatology, Endocrinology, Rheumatology and Infectious Diseases, Department of Internal Medicine I, University Hospital Regensburg, Regensburg, Germany; Gastroenterology, Hepatology, Endocrinology, Rheumatology and Infectious Diseases, Department of Internal Medicine I, University Hospital Regensburg, Regensburg, Germany; Gastroenterology, Hepatology, Endocrinology, Rheumatology and Infectious Diseases, Department of Internal Medicine I, University Hospital Regensburg, Regensburg, Germany; Gastroenterology, Hepatology, Endocrinology, Rheumatology and Infectious Diseases, Department of Internal Medicine I, University Hospital Regensburg, Regensburg, Germany; Gastroenterology, Hepatology, Endocrinology, Rheumatology and Infectious Diseases, Department of Internal Medicine I, University Hospital Regensburg, Regensburg, Germany; Gastroenterology, Hepatology, Endocrinology, Rheumatology and Infectious Diseases, Department of Internal Medicine I, University Hospital Regensburg, Regensburg, Germany; Gastroenterology, Hepatology, Endocrinology, Rheumatology and Infectious Diseases, Department of Internal Medicine I, University Hospital Regensburg, Regensburg, Germany; Gastroenterology, Hepatology, Endocrinology, Rheumatology and Infectious Diseases, Department of Internal Medicine I, University Hospital Regensburg, Regensburg, Germany; Gastroenterology, Hepatology, Endocrinology, Rheumatology and Infectious Diseases, Department of Internal Medicine I, University Hospital Regensburg, Regensburg, Germany; Gastroenterology, Hepatology, Endocrinology, Rheumatology and Infectious Diseases, Department of Internal Medicine I, University Hospital Regensburg, Regensburg, Germany; Department of Medicine B—Gastroenterology and Hepatology, University Hospital Münster, Münster, Germany; Practice for Internal Medicine, Münster, Germany; Department of Medicine I, University Medical Center Schleswig-Holstein, Campus Lübeck, Lübeck, Germany; Department of Medicine I, University Medical Center Schleswig-Holstein, Campus Lübeck, Lübeck, Germany; Department of Medicine I, University Medical Center Schleswig-Holstein, Campus Lübeck, Lübeck, Germany; Institute of Chemistry and Metabolomics, University of Lübeck, Lübeck, Germany; Institute of Nutritional Medicine, University Medical Center Schleswig-Holstein, Campus Lübeck, Lübeck, Germany; Department of Medicine I, University Medical Center Schleswig-Holstein, Campus Lübeck, Lübeck, Germany; Institute of Nutritional Medicine, University Medical Center Schleswig-Holstein, Campus Lübeck, Lübeck, Germany; Department of Medicine I, University Medical Center Schleswig-Holstein, Campus Lübeck, Lübeck, Germany; Fraunhofer Research Institution for Individualized and Cell-Based Medical Engineering, Lübeck, Germany; Gastroenterology, Hepatology, Endocrinology, Rheumatology and Infectious Diseases, Department of Internal Medicine I, University Hospital Regensburg, Regensburg, Germany

**Keywords:** lipoprotein, apolipoprotein, metabolomics, IBD, amino acids, nuclear magnetic resonance spectroscopy, biomarker

## Abstract

**Background:**

Accurate biomarkers for disease activity and progression in patients with inflammatory bowel disease (IBD) are a prerequisite for individual disease characterization and personalized therapy. We show that metabolic profiling of serum from IBD patients is a promising approach to establish biomarkers. The aim of this work was to characterize metabolomic and lipidomic serum profiles of IBD patients in order to identify metabolic fingerprints unique to the disease.

**Methods:**

Serum samples were obtained from 55 patients with Crohn’s disease (CD), 34 patients with ulcerative colitis (UC), and 40 healthy control (HC) individuals and analyzed using proton nuclear magnetic resonance spectroscopy. Classification of patients and HC individuals was achieved by orthogonal partial least squares discriminant analysis and univariate analysis approaches. Disease activity was assessed using the Gastrointestinal Symptom Rating Scale.

**Results:**

Serum metabolome significantly differed between CD patients, UC patients, and HC individuals. The metabolomic differences of UC and CD patients compared with HC individuals were more pronounced than the differences between UC and CD patients. Differences in serum levels of pyruvic acid, histidine, and the branched-chain amino acids leucine and valine were detected. The size of low-density lipoprotein particles shifted from large to small dense particles in patients with CD. Of note, apolipoprotein A1 and A2 serum levels were decreased in CD and UC patients with higher fecal calprotectin levels. The Gastrointestinal Symptom Rating Scale is negatively associated with the concentration of apolipoprotein A2.

**Conclusions:**

Metabolomic assessment of serum samples facilitated the differentiation of IBD patients and HC individuals. These differences were constituted by changes in amino acid and lipoprotein levels. Furthermore, disease activity in IBD patients was associated with decreased levels of the atheroprotective apolipoproteins A1 and A2.

**Key Messages**

Biomarkers for diagnosing the activity and remission of inflammatory bowel disease (IBD) are urgently needed because IBD increases the risk of atherosclerosis, emphasizing the importance of biomarkers to identify patients at high risk for timely intervention. Active IBD is linked to reduced levels of atheroprotective apolipoproteins A1 and A2, along with a shift from large to small low-density lipoprotein particles. Our data indicate potential circulating apolipoproteins and small dense low-density lipoprotein that may emerge as new biomarkers for IBD activity and IBD-associated atherosclerosis.

## Introduction

Inflammatory bowel disease (IBD) is a global health challenge with increasing incidence, especially in countries with Western lifestyle.^1^ In the 21st century, prevalence of IBD exceeded 0.3% of the total population in Western countries, such as Canada, Denmark, Germany, Hungary, Australia, New Zealand, Sweden, the United Kingdom, and the United States.^2^ Pathogenesis of Crohn’s disease (CD) and ulcerative colitis (UC) is multifactorial and associated with various risk factors including environmental parameters, dietary habits, several genetic variants, dysregulated immune response, and qualitative and quantitative deranged gut microbiota.^3,4^

Diagnosis of IBD and differentiation between CD and UC rely on a time-intensive, human resource–intensive, and cost-intensive multidisciplinary approach, which requires integration and evaluation of patients’ history, microbiology, endoscopy, imaging, hematology, and histology.^5,6^ Despite complex diagnostic procedures, approximately 10% of IBDs cannot be assigned to a specific entity.^7^ However, accurate classification of IBD patients is important for patient management and therapy. In clinical practice, there is currently a lack of simple and specific biomarkers to assess disease activity, remission, or progression. In addition, there is currently no biomarker available that predicts therapeutic response upon IBD-specific therapy. Therefore, it is essential to identify biomarkers that improve understanding of disease pathophysiology to further identify severe courses and ultimately for personalized therapy decisions for IBD patients.

Unbiased surveys of the serum metabolome have the potential to reveal novel biomarkers for disease activity and mediators of disease pathology.^8-10^ Knowledge of a unique metabolic and lipidomic fingerprint in IBD patients could constitute a clinically relevant tool for diagnosis, treatment, and detection of disease mechanisms.^11-13^ Previously, metabolic pilot studies identified potential biomarkers that may be appropriate for the stratification of IBD patients but await confirmation. Particularly, differences in choline, amino acid, and lipid metabolites in IBD patients compared with healthy control (HC) individuals have been reported.^12-14^

Patients with IBD experience dyslipidemia and lipid abnormalities that correlate with disease activity.^15,16^ Accordingly, IBD is associated with an increased risk of atherosclerotic cardiovascular disease (CVD) and venous thromboembolism.^17-20^ Disease activity and distinct incident patterns in IBD correlate with a high risk for atherosclerotic CVD and venous thromboembolism.^21^ Therefore, profiling of lipid and cholesterol metabolism in IBD is of particular clinical interest. Previous studies on lipid abnormalities in IBD patients comparing serum triglyceride levels were described for IBD patients and HC individuals.^22^ In a large study of 393 IBD patients, decreased total and high-density lipoprotein (HDL) cholesterol levels and increased low-density lipoprotein (LDL) cholesterol levels were observed.^23^ Although several other studies demonstrated abnormalities in cholesterol and lipoproteins, these changes were not consistently observed in all studies.^22^ Thus, to analyze disorders of lipid metabolism in IBD patients, a more refined approach is required beyond the determination of total cholesterol and lipoprotein levels.

In general, lipoproteins are composed of lipids and apolipoproteins. HDL, LDL, intermediate-density lipoprotein, and very LDL (VLDL) differ in density, size, and composition. For each of these lipoprotein classes, several subclasses can be identified via nuclear magnetic resonance (NMR) spectroscopy that vary in their atherogenic properties.^24,25^ While the numbering of subclasses is not standardized, the system established by the Bruker in vitro diagnostic research (IVDr) protocol is most commonly used. In this protocol, 5 VLDL, 6 LDL, and 4 HDL subclasses can be distinguished.^26^ Among these, density increases to higher subclasses. For LDL, the particles of higher density particularly contribute to CVDs.^27,28^ HDL is essential for reverse cholesterol transport and is associated with protective capacities against CVD. Small HDL particles and lipid-poor particles have a high capacity for ABCA1 (ATP-binding cassette transporter A1)-mediated cholesterol efflux and act as anti-inflammatory factors. Larger HDL particles function as antioxidants.^29^ Apolipoprotein A1 (ApoA1) is found in all and ApoA2 in approximately 75% of the circulating HDL particles.^30^

Thus, the aim of the current project was to analyze metabolic and lipoprotein serum profiles of IBD patients with particular emphasis on lipoprotein subclasses and their components using NMR spectroscopy. In this regard, we aimed to identify potential novel biomarkers associated with IBD pathology, disease severity, diagnosis, and treatment response.

## Methods

### Ethical Considerations

The study was approved by the Ethics Committees of the Universities of Lübeck and Regensburg (Protocol No. 21-2390-101 and No. 22-104), and all participants gave written informed consent to the study.

### Study Subjects

Patients with confirmed diagnosis of CD (n = 55) or UC (n = 34) were recruited from the outpatient department or from the inpatient setting at the university hospitals of Regensburg and Lübeck. IBD diagnosis was established by an IBD specialist using accepted clinical, endoscopic, and histologic criteria^31-33^ and following the current guidelines.^34^ Individuals who were pregnant, had known coagulopathy, had prior organ transplantations, or were unable to give informed consent were not enrolled. Patients were asked to present to the clinic for blood sampling in a fasting condition.

HC individuals with no previous medication, chronic diseases, or pregnancy were recruited from the LuMeR (Lübeck Metabolomics Reference) cohort. This cohort was developed as part of the ELISA (Lübeck Longitudinal Investigation of SARS-CoV-2 Infection) framework.^35^ The HC cohort was matched for age, body mass index (BMI), and sex (n = 40).

For all IBD patients, demographic data including age, sex, medical history, and current medication were documented, and disease status was evaluated on the day of serum collection. Medical history included possible gastrointestinal tract surgery, previous tumor diseases and/or immunosuppression, and comorbidities such as diabetes mellitus and/or arterial hypertension. Medication history included previous and current IBD-specific therapy. Disease progression was categorized as steroid-sensitive, steroid-dependent, or steroid-refractory inflammatory flair of the underlying disease.^36^ Possible extraintestinal manifestations such as primary sclerosing cholangitis and/or arthropathies, cutaneous manifestations, or ocular involvement were evaluated. For each individual patient, the localization of IBD in the gastrointestinal tract was recorded. At the time of serum collection, weight and height of the patients were determined and BMI was calculated. With reference to BMI, IBD patients were divided into underweight, normal weight, and overweight (3 subgroups of overweight). For BMI, data were not collected if they had not been available for more than 12 months.

General well-being, abdominal pain, and the number of stools per day were assessed by participants using the Gastrointestinal Symptom Rating Scale (GSRS).^37^ Among the included patients, endoscopy data that were closest in time to serum collection were used and endoscopic activity scores were documented. Endoscopies with a latency of >6 weeks to serum collection were excluded. For CD, the Simple Endoscopic Score for Crohn’s Disease (SES-CD) was applied, and for UC, the Ulcerative Colitis Endoscopic of Severity (UCEIS) was applied. Here, the definition of endoscopic healing was adjusted to the current STRIDE-II (Selecting Therapeutic Targets in Inflammatory Bowel Disease II) consensus.^38^ For CD, SES-CD <3 points or absence of ulceration was classified as endoscopic healing. In UC, UCEIS ≤1 points was considered as endoscopic healing.

In summary, we recorded different surrogate markers to objectify current disease activity: a clinical activity score (GSRS), endoscopic scores (UCEIS, SES-CD), serologic parameters (C-reactive protein), and the fecal calprotectin as a noninvasive surrogate parameter. Calprotectin values were only included if they had been determined ±1 month around study inclusion.

### Analysis of Human Serum Metabolites

Serum samples were analyzed by proton NMR spectroscopy using Bruker’s standardized IVDr procedure (Bruker BioSpin). The protocol was described previously.^39,40^ In brief, frozen aliquots were thawed at room temperature for several minutes. Serum and phosphate buffer (75 mM, pH 7.4) were homogenized by manual panning and 600 µL were transferred to a 5 mm NMR tube. Tubes were cooled at 279 K in an automated SampleJet (Bruker) until measurement. Samples were analyzed at a 600 MHz Avance III HD NMR spectrometer (Bruker) with TXI probe at 310 K. A standard operating procedure was performed prior to analysis, including the check of temperature calibration, quantification, and water suppression performance.

The 1-dimensional NOESY experiment (pulse program: noesygppr1d) and 1-dimensional Carr-Purcell-Meiboom-Gill spin-echo experiment (pulse program: cpmgpr1d) for the suppression of proteins and other macromolecular signals were recorded per sample. Bruker Quantification in plasma/serum (B.I.Quant-PS 2.0.0) and Bruker IVDr Lipoprotein Subclass Analysis (B.I.-LISA) were used to automatically quantify 39 metabolites (+2 technical additives) and 112 lipoprotein parameters (Bruker BioSpin). Lipoprotein parameters contain several subfractions of cholesterol, free cholesterol, phospholipids (PL), triglycerides (TG), and Apo.

### Statistical Analysis

Univariate and multivariate methods were used to analyze quantitative IVDr data. For multivariate partial least squares discriminant analysis, the PLS toolbox was used (Eigenvector Research, Inc). Data were variance-scaled and mean-centered, and the model was orthogonalized. The orthogonal partial least squares discriminant analysis (OPLS-DA) was calculated followed by cross-validation using venetian blinds and the area under the receiver-operating characteristic curve (AUROC) was computed. The robustness of the model was further evaluated by a permutation test with 100 iterations. For univariate analyses, unpaired *t* tests adjusted for multiple comparisons by the method of Benjamini, Krieger, and Yekutieli with a false discovery approach were applied. Univariate analyses as well as simple linear regression analyses were performed in GraphPad Prism 9.4.0 (GraphPad Software).

## Results

### Cohort Demographics

Demographic data of all patients were collected. Clinical characterization of the cohort was performed in order to determine current disease activity and phenotype of the disease as well as to document the current therapy (Table 1).

**Table 1.** Demographics and characteristics of IBD patients in the cohort.

	Patients with CD (n = 55)	Patients with UC (n = 34)
Median age, y	40	44
Sex		
Male	24 (44)	20 (59)
Female	31 (56)	14 (41)
Weight (BMI)		
Underweight (<18.5 kg/m^2^)	3 (5)	4 (12)
Normal weight (18.5-24.9 kg/m^2^)	17 (31)	13 (38)
Overweight (25-29.9 kg/m^2^)	14 (25)	6 (18)
Obese class I (30-34.9 kg/m^2^)	10 (18)	7 (21)
Obese class II (35-39.9 kg/m^2^)	2 (4)	0 (0)
Obese class III (>40 kg/m^2^)	0 (0)	0 (0)
Missing data	9 (16)	4 (12)
Median BMI, kg/m^2^	26	24
Mean BMI, kg/m^2^	26 (5)	25 (5)
Median age at initial diagnosis, y	23	25
Mean age at initial diagnosis, y	26 ± 11	30 ± 13
Manifestation site		
Multilocular Crohn	46 (84)	0 (0)
Mouth	1 (2)	0 (0)
Upper gastrointestinal tract	13 (24)	0 (0)
Small intestine	20 (36)	0 (0)
Ileocecal region	48 (87)	0 (0)
Ascending colon	30 (55)	0 (0)
Transverse colon	24 (44)	0 (0)
Descending colon	23 (42)	0 (0)
Sigmoid colon	26 (47)	0 (0)
Proctitis	28 (51)	2 (6)
Proctosigmoiditis	0 (0)	4 (12)
Left-sided colitis	0 (0)	5 (15)
Pancolitis	0 (0)	18 (53)
Pancolitis with backwash ileitis	0 (0)	4 (12)
Nonspecific colon involvement	4 (7)	0 (0)
Missing data	0 (0)	1 (3)
Extraintestinal manifestation		
Skin involvement	14 (25)	6 (18)
Arthralgia	20 (36)	5 (15)
Eye involvement	8 (15)	1 (3)
Primary sclerosing cholangitis	1 (2)	5 (15)
None	24 (44)	21 (62)
Concomitant disease		
Hypertension	7 (13)	7 (21)
Diabetes mellitus	1 (2)	0 (0)
None of the above	47 (85)	27 (79)
Endoscopic healing		
Yes	20 (36)	12 (35)
No	30 (55)	15 (44)
Missing data	5 (9)	7 (21)
GSRS		
None (13)	4 (7)	2 (6)
Minor complaints (14-39)	29 (53)	16 (47)
Moderate complaints (40-65)	19 (35)	8 (24)
Strong complaints (66-91)	0 (0)	2 (6)
Missing data	3 (5)	6 (18)
Calprotectin		
≤50 µg/g	23 (42)	12 (35)
≤150µg/g	15 (27)	7 (21)
>150 µg/g	6 (11)	4 (12)
≥500 µg/g	4 (7)	8 (24)
Missing data	7 (13)	3 (9)
Medication at the time of sample collection		
Anti-TNF monotherapy	17 (31)	2 (6)
Anti-TNF + mesalazine	2 (4)	1 (3)
Anti-TNF + azathioprine	2 (4)	0 (0)
Anti-TNF + corticosteroids	4 (7)	1 (3)
Anti-TNF + ustekinumab	1 (2)	0 (0)
Anti-TNF + corticosteroids + methotrexate	1 (2)	0 (0)
Ustekinumab monotherapy	13 (24)	1 (3)
Ustekinumab + mesalazine	2 (4)	1 (3)
Ustekinumab + corticosteroids	0 (0)	3 (9)
Ustekinumab + mesalazine + corticosteroids	2 (4)	2 (6)
Vedolizumab monotherapy	1 (2)	2 (6)
Vedolizumab + mesalazine	0 (0)	2 (6)
Vedolizumab + mesalazine + corticosteroids	0 (0)	2 (6)
Vedolizumab + mesalazine + azathioprine	0 (0)	1 (3)
Tofacitinib monotherapy	0 (0)	0 (0)
Mesalazine monotherapy	3 (5)	6 (18)
Azathioprine monotherapy	0 (0)	1 (3)
Methotrexate monotherapy	0 (0)	0 (0)
Corticosteroids monotherapy	2 (4)	1 (3)
None	3 (5)	3 (9)

Values are n (%), unless otherwise indicated.

Abbreviations: BMI, body mass index; CD, Crohn’s disease; GSRS, Gastrointestinal Symptom Rating Scale; TNF, tumor necrosis factor; UC, ulcerative colitis;

A total of 89 patients with confirmed diagnosis of IBD and 40 healthy sex-, age-, and BMI-matched control individuals were included in our study (Supplementary Table 1). In the studied IBD cohort, detailed phenotyping of the patients was performed and the disease course including comorbidities, complications, and drug therapies was recorded. The data presented in Table 1 provide an overview of the disease phenotype at initial diagnosis, current disease activity, and therapy. The median age of patients with CD was 42 years, and that of patients with UC was 41 years. A total of 38% of patients with UC and 31% of patients with CD presented with normal body weight. In 62% of patients experiencing UC, the entire colon was affected in form of pancolitis. History of intestinal surgery, extraintestinal manifestations, and the number of relapses was recorded to assess disease severity and progression. There was a high proportion of extensive gastrointestinal involvement, with 53% with pancolitis, 12% with pancolitis and reflux ileitis for patients with UC, and 84% with multilocular CD. Endoscopic activity was detected in 56% with UC and in 45% with CD. Together, these data indicate pronounced disease activity in the studied cohort.

### Global Metabolic Profiles of Patients With IBD and HC Individuals

**Figure 1.**
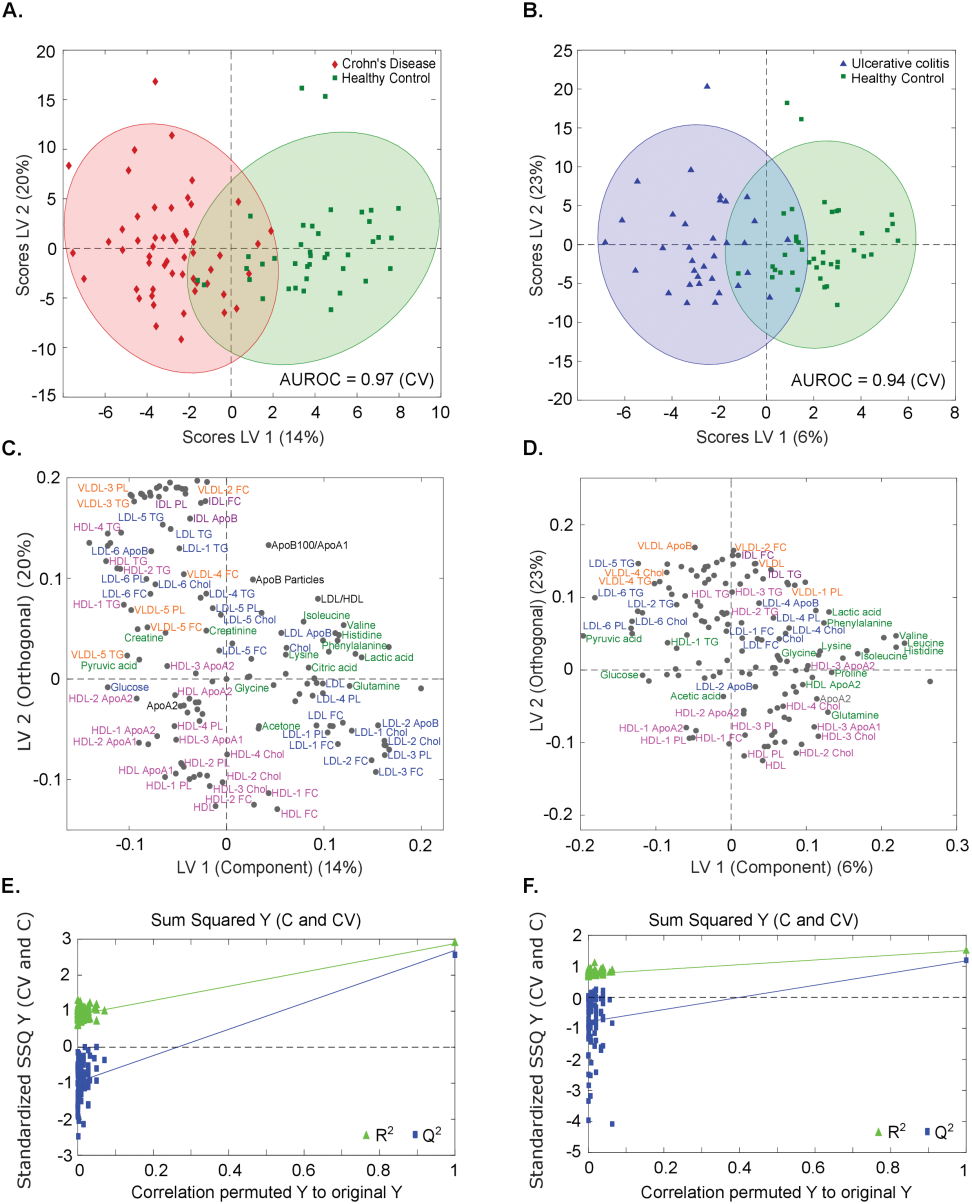
Metabolic profiles of patients with Crohn’s disease (CD) (left) and ulcerative colitis (UC) (right panel) compared with healthy control (HC) individuals. A and B, Scores plot of orthogonal partial least square discriminant analysis (OPLS-DA) for nuclear magnetic resonance data quantified by Bruker’s in vitro diagnostic research show a distinct separation between CD (red) and HC (green) individuals as well as UC (blue) and HC (green) individuals. The area under the receiver-operating characteristic curve (AUROC) for cross-validation (CV) over 90% illustrates very good classification models for both comparisons. C and D, Color-highlighted loadings plots show metabolites (dark green) and lipid parameters (very low-density lipoprotein [VLDL]: orange; intermediate-density lipoprotein [IDL]: purple; low-density lipoprotein [LDL]: blue; high-density lipoprotein [HDL]: pink) contributing to this separation. E and F, The quality of the models was evaluated by permutation testing. Apo, apolipoprotein; Chol, cholesterol; FC, free cholesterol; LV, latent variable; PL, phospholipids; SSQ, sum of squares; TG, triglycerides.

After clinical characterization of the study cohort, a global metabolomic examination of patients and control individuals was performed to evaluate whether a distinct metabolomic profile could be attributed to CD or UC. Adequately matched HC individuals served as the control group (Supplementary Table 1). Serum NMR IVDr data of the 2 IBD cohorts were analyzed with a multivariate OPLS-DA and were compared with data of HC individuals (Figure 1).

Figure 1A, C, and E (left) display scores and loadings plots as well as permutation analysis of OPLS-DA for the comparison between CD patients (red) and HC individuals (green). A very good separation between CD patients and HC individuals by metabolomic and lipoprotein profiling was observed in the scores plot. The discrimination was very efficient, as evidenced by the AUROC value of 97% (Figure 1A). The loadings plot indicated that alterations of the content of several amino acids (histidine, leucine, isoleucine) and keto acids such as pyruvic acid, citric acid, and lactic acid were partly responsible for the good separation. Furthermore, VLDL and LDL levels, as well as the lipid composition of VLDL, LDL, and HDL, differed between the groups (Figure 1C).

Similarly, patients with UC (blue) were compared with the same HC cohort (green). Comparison of the metabolome of UC and HC individuals revealed a good separation between the groups and a very good model with an AUROC for cross-validation of 94% (Figure 1C). Not only LDL parameters, but also VLDL fractions and metabolites were responsible for the separation of both groups according to the loadings plot (Figure 1D). The quality of both models was evaluated by permutation tests (Figure 1E, F).

### Metabolic Profile of Patients With IBD and HC Individuals

**Figure 2.**
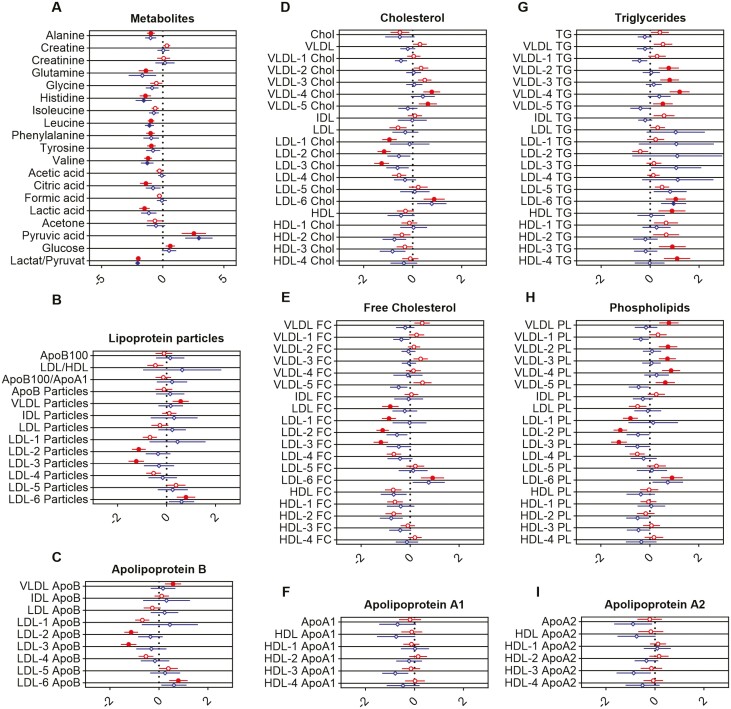
Forest plots showing changes of specific metabolites (A) and lipoprotein classes (B-I) in serum of patients with Crohn’s disease (CD) and ulcerative colitis (UC). The dotted center line indicates the reference average of healthy control (HC) individuals, whereas circles and diamonds on horizontal axes show the relative deviation normalized to the standard deviation of HC individuals. Red circles show changes for CD patients and blue diamonds for UC patients, both with matched HC individuals as reference (vertical line). Statistically significant differences between CD and UC patients and HC individuals determined using the false discovery method of Benjamini, Krieger, and Yekutieli (Q = 1%) are indicated by filled circles or diamonds. Apo, apolipoprotein; Chol, cholesterol; FC, free cholesterol; HDL, high-density lipoprotein; IDL, intermediate-density lipoprotein; LDL, low-density lipoprotein; PL, phospholipids; TG, triglycerides; VLDL, very low-density lipoprotein.

Subsequently, specific metabolites such as amino acids and lipoproteins quantified by the IVDr protocol were examined in detail and differences between patients with CD or UC and HC individuals were analyzed (Figure 2, Supplementary Table 2). The forest plot in Figure 2 shows the relative deviation of CD (red) and UC (blue) patients from HC individuals normalized to the standard deviation of HC individuals. The dashed vertical line represents HC individuals as a reference.

A significant decrease in the content of alanine, glutamine, histidine, leucine, phenylalanine, tyrosine, and valine, as well as of citric acid, lactic acid, and the ratio between lactic and pyruvic acid, was observed in the serum of CD patients. Higher serum concentrations were noted for glucose and pyruvic acid in particular. Similar alterations of these metabolites were also observed in UC. Of particular note, none of these factors differed between CD and UC (Figure 2).

### Lipidomic Profile of Patients With IBD and HC Individuals

To determine the lipoprotein profiles of IBD, lipoprotein compositions of patients with CD were compared with HC samples. CD patients displayed increased levels of VLDL particles, VLDL ApoB, and VLDL PL (Figure 2B, C, and H). VLDL-4 and VLDL-5 showed higher cholesterol, and VLDL-2 to VLDL-5 increased TG and PL levels in patients with CD (Figure 2G, H). Significantly decreased levels of the subfractions LDL-2 and LDL-3 particles, as well as LDL-2 and LDL-3 ApoB, were observed. Interestingly, LDL-6 particles and LDL-6 ApoB were increased, revealing a shift of LDL from larger to very small particles (Figure 2B, C). Accordingly, LDL free cholesterol and the subfractions LDL-1, LDL-2, and LDL-3 of (free) cholesterol as well as PL levels were reduced in the serum of patients with CD compared with HC individuals (Figure 2D, E, and H). TG concentrations of these LDL subfractions were not altered (Figure 2G). As observed for LDL-6 particles, cholesterol, free cholesterol, PL, and TG were significantly increased in CD (Figure 2D, E, G, and H). ApoA1 and ApoA2 were similar in CD and HC individuals (Figure 2F, I). In contrast, HDL TG and the subfractions HDL-3 and HDL-4 TG were increased (Figure 2G).

Lipid profile changes were much less pronounced in UC patients compared with HC individuals. Similar to patients with CD, UC patients showed a trend for increased LDL parameters, but only LDL-6 TG displayed a statistically significant elevation. Notably, TG bound in all LDL subfractions were slightly increased in UC patients compared with HC individuals. For larger-sized LDL parameters (LDL-1 to LDL-3), only a tendency for reduction was observed.

### Lipidomic Profile of Patients With CD and UC

**Figure 3.**
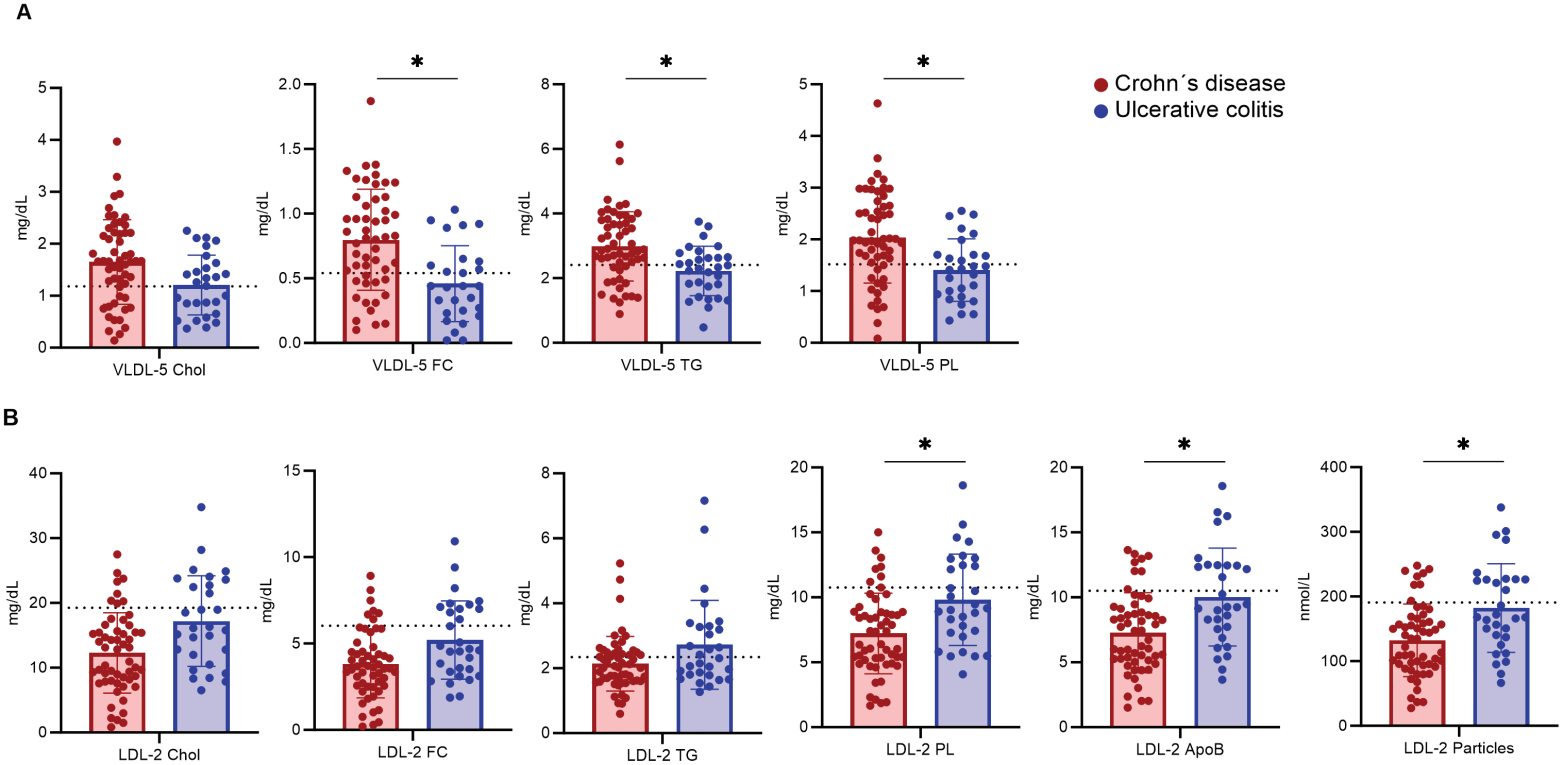
Very low-density lipoprotein-5 (VLDL-5) and low-density lipoprotein-2 (LDL-2) parameters differ between patients with Crohn’s disease and ulcerative colitis. Significant changes were determined by multiple unpaired *t* test using the false discovery method of Benjamini, Krieger, and Yekutieli (Q = 5%). The dashed line represents the mean value of healthy control individuals. Apo, apolipoprotein; Chol, cholesterol; FC, free cholesterol; PL, phospholipids; TG, triglycerides.

Differences in the lipidomic profiles of CD compared with UC patients were analyzed. Alterations between both disease cohorts were found for VLDL-5 parameters (Figure 3A) and LDL-2 parameters (Figure 3B). Patients with CD showed significantly elevated VLDL-5 TG and VLDL-5 PL compared with patients with UC (Figure 3A). In contrast, LDL parameters were reduced in CD compared with UC patients with significant changes for LDL-2 PL, LDL-2 ApoB, and LDL-2 particles (Figure 3B).

Taken together, distinct differences were identified in VLDL-5 and LDL-2 subfractions between CD and UC patients.

### Lipidomic Parameters Associate With Clinical Features of Disease Activity

To analyze possible associations of disease severity and metabolomic/lipidomic markers, calprotectin levels and GSRS scores were correlated with NMR parameters. Fecal calprotectin is an important noninvasive surrogate marker for the evaluation of mucosal inflammation.^41,42^ The GSRS objectifies the clinical symptoms of discomfort.^37^

**Figure 4.**
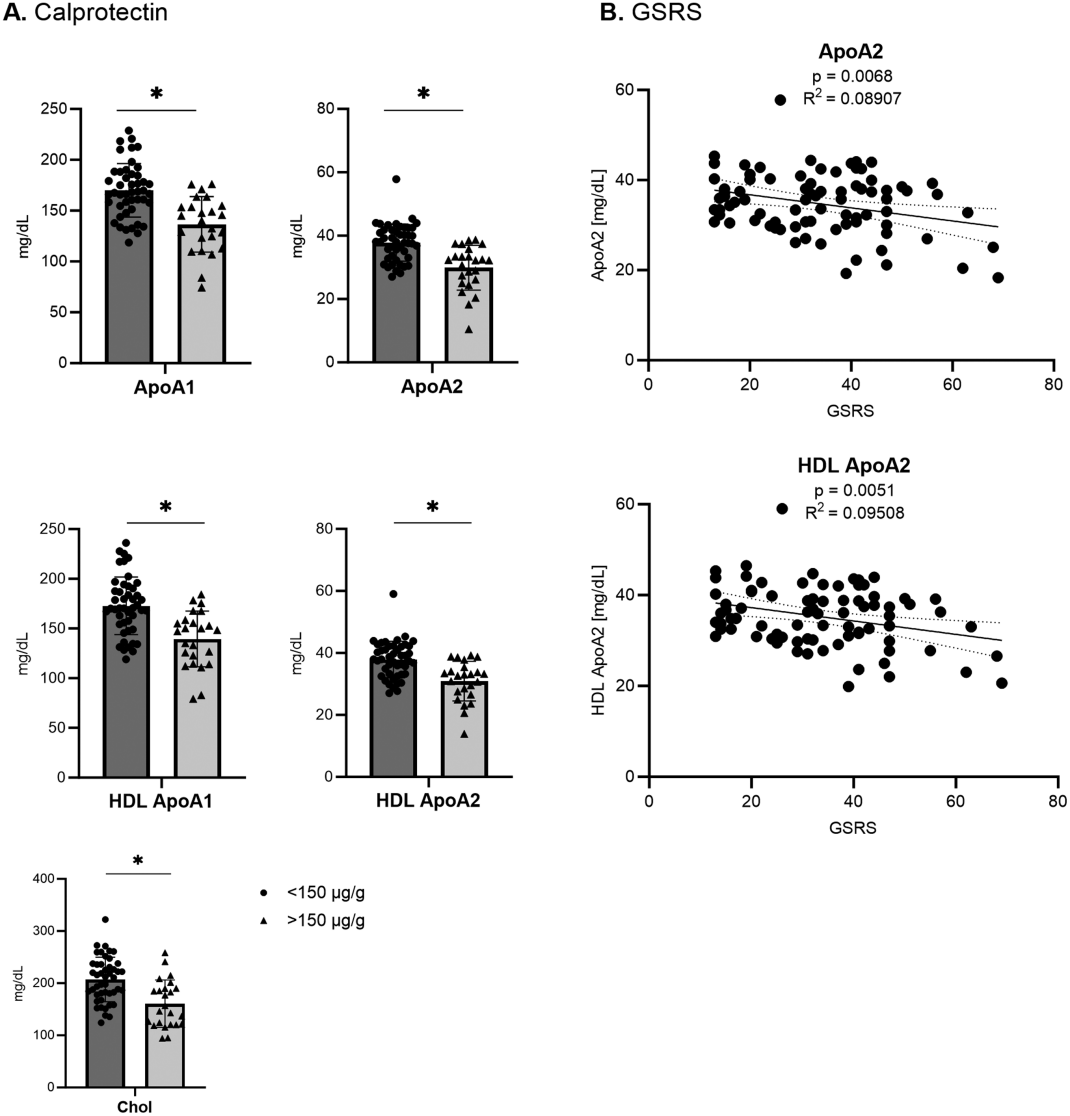
Associations between clinical markers of patients with inflammatory bowel disease and apolipoproteins (Apo). A, Significant changes determined by multiple unpaired *t* test using the false discovery method of Benjamini, Krieger, and Yekutieli (Q = 1%) are shown for patients with low (<150 µg/g) and high (>150 µg/g) calprotectin levels. (B) Associations of Gastrointestinal Symptom Rating Scale (GSRS) with ApoA2 and high-density lipoprotein (HDL) ApoA2. Chol, cholesterol.

As a result, a significant reduction of ApoA1 and ApoA2 and their HDL fractions as well as cholesterol in patients with high fecal calprotectin levels was observed (Figure 4A). Furthermore, ApoA2 showed a negative relationship with GSRS, indicating an association of low ApoA2 levels with increased disease severity (Figure 4B).

In summary, we identified a metabolic signature in IBD patients that included decreased levels of several amino acids and increased levels of pyruvic acid. Furthermore, we observed a shift of size and density in LDL particles toward atherogenic subclasses. Finally, disease activity, assessed by calprotectin levels and/or GSRS, was associated with reduced apolipoprotein A1 and A2 levels.

## Discussion

Metabolic alterations are frequently observed in patients with IBD, but their metabolic and lipoprotein profiles remain poorly characterized. New technical methods allow a more in-depth analysis of lipoproteins, their subclasses, and bound lipids. To the best of our knowledge, we presented metabolic and lipoprotein signature analysis by NMR spectroscopy of the largest cohort of IBD patients and HC individuals to date. Our comprehensive studies have shown that the metabolomic and lipidomic profiles of CD and UC patients differ significantly from those of HC individuals and that cardiovascular risk factors can be defined.

Specific clinical criteria make a severe, protracted course of disease more likely and require early use of biologicals. Such criteria include extensive inflammation at multiple sites, young age at first diagnosis, and steroid-refractory disease.^43,44^ Peyrin-Biroulet et al^44^ proposed to define the severity of the IBD disease. Their work distinguishes 3 main areas relevant to the assessment of disease severity in IBD: impact of the disease on the patient, disease burden, and disease progression.^44^ These 3 aspects were used as a guide to define criteria for disease severity in our study population.^44^ In our study, the impact of the disease on the patient was objectified using the GSRS.^37^ For disease burden and disease progression, serum C-reactive protein, fecal calprotectin, disease distribution, and macroscopic activity determined by endoscopy were considered.

A central goal of our work was to establish a metabolomic profile for patients with different disease phenotypes of IBD. Our results (Figures 1 and 2) show clear differences in the metabolomic profile of patients with IBD compared with HC individuals. This is consistent with previous literature.^9,13,45,46^ Bjerrum et al^47^ detected significant differences in metabolic profiles that allowed differentiation between active IBD patients and control individuals as well as between CD and UC patients. Metabolites with differential significance primarily belonged to a number of amino acids and microbiota-related short-chain fatty acids.^47^ This is consistent with our data. In contrast to Bjerrum et al, we performed serum analyses, whereas Bjerrum et al conducted stool examinations. Stephens et al^13^ described a distinct metabolomic profile of patients with IBD compared with HC individuals, analogous to our work. In this work, urinary metabolomics was used to distinguish patients with IBD from healthy people. Key differences between IBD and healthy individuals included tricarboxylic acid cycle intermediates, gut microflora metabolites, and amino acids. Comparison of CD and UC patients showed discrimination, but elimination of patients with surgery as a confounding factor showed that CD could not be distinguished from UC.^13^ Scoville et al^48^ demonstrated that a number of amino acid–related, lipid-related, and tricarboxylic acid cycle–related metabolites were significantly altered in IBD patients, more specifically in CD patients. Accordingly, changes of the metabolome were more evident in CD than UC in the current cohort. In the work of Williams et al,^12^ CD and UC cohorts differed from the control cohort by increased levels of serum VLDL cholesterol, reduced LDL and HDL cholesterol and choline, and increased lactic acid and *N*-acetylated glycoprotein. While we were able to confirm the reduced LDL cholesterol in the IBD cohort, reduced HDL cholesterol and increased lactic acid levels are divergent to our data. Particularly, elevated serum levels of lactic acid appear to be a potential biomarker in IBD patients. Increased concentrations have been demonstrated in several well-controlled studies conducted by our research group and other teams.^49^ However, this could not be demonstrated in this study cohort (see Limitations).

Likewise, changes in branched-chain amino acids (BCAAs) such as leucine, isoleucine, and valine have been consistently observed in CD and UC patients compared with HC individuals.^45,50,51^ Valine levels are apparently lower in CD and UC.^46,52^ Scoville et al^48^ and Diab et al^53^ confirmed a similar trend for leucine and showed that IBD is associated with low leucine concentration. We confirmed the trends for valine and leucine in our cohort. Furthermore, there is a trend for reduced isoleucine levels in IBD patients compared with HC individuals in our cohort as well (Figure 2A). To date, changes in BCAAs have been well studied, particularly in insulin resistance.^54^ However, BCAA changes in IBD and their clinical implications remain less clear. It is conceivable that these variations/alterations could be an expression of chronic inflammation and altered composition of the gut microbiome. Lower glutamine concentrations were found in both patients with CD and UC when compared with control subjects.^48,55^ This observation was confirmed in our cohort (Figure 2A). Glutamine plays an important role in intestinal integrity by regulating tight junction proteins and preventing bacterial translocation. Reduced plasma glutamine concentration has also been associated with increased immune activation.^56^ Similarly, decreased serum histidine concentration has been measured in IBD patients.^45,55^ Low histidine concentration turned out to be a prognostic marker for an increased risk of acute relapse after 6 months^45^ and 1 year.^57^ The reduced serum histidine concentration in IBD patients was confirmed in our analyses (Figure 2A). Moreover, we observed a significant increase in pyruvic acid for patients with CD and UC compared with HC individuals in our cohort (Figure 2A). This observation fits with the pathophysiological consideration that pyruvic acid plays a crucial role in the regulation of immune modulation in the gut. It is known that metabolites such as pyruvic acid, dietary components, xenobiotics, or chemicals can activate the aryl hydrocarbon receptor and trigger the modulation of inflammatory responses. Pro- and anti-inflammatory signal pathways are regulated via the aryl hydrocarbon receptor,^58^ indicating potential pathophysiological pathways mediated/regulated by the identified metabolites.

Furthermore, lipid profiles and in particular the lipid subclasses of the studied patients with UC and CD were examined and compared with HC individuals. There is emerging evidence of a link between systemic inflammation and low serum LDL concentration (eg, in the context of sepsis or in autoinflammatory diseases such as rheumatoid arthritis or familial Mediterranean fever).^59-61^ In addition, serum LDL concentrations were reduced in subjects just recovering from mild bacterial infection, paralleling elevated acute phase reaction markers.^62^ The hypothesis that systemic inflammation may cause the observed decrease in LDL cholesterol in these disease entities is supported by the recovery of LDL cholesterol toward the normal range upon treatment of rheumatoid arthritis or ankylosing spondylitis with anti-inflammatory drugs.^63,64^ Furthermore, experimental induction of acute phase reaction in humans by intravenous administration of low doses of endotoxin reproducibly resulted in a transient decrease in LDL cholesterol within 12 hours before LDL cholesterol slowly recovered to normal levels over the following 24 to 48 hours.^59,65^ Referring to these pathophysiological considerations, we observed reduced LDL serum concentrations in our studied IBD cohort, especially in CD patients (Figure 2). However, there are other gastrointestinal and systemic conditions (such as *Helicobacter pylori* colonization of the stomach, celiac disease, or psoriatic arthritis) associated with increased LDL serum concentration.^66-68^ Moreover, increased LDL cholesterol levels have been described in patients with irritable bowel syndrome, leaving LDL cholesterol on its own unspecific for IBD.^69^ Previous pathophysiological considerations linking mechanisms directly involving intestinal inflammation to lipoprotein metabolism focus on the secretion of chylomicrons by enterocytes. Studies in hamsters have shown that infusion of tumor necrosis factor leads to increased ApoB-48 secretion by enterocytes.^70,71^ It has been postulated that the majority of excess cholesterol is secreted by the liver and that hepatocytes are therefore the critical cell type regulating systemic LDL cholesterol levels. Recent findings suggest that approximately 35% of excreted cholesterol is delivered directly into the intestinal lumen via enterocytes through a process known as transintestinal cholesterol efflux.^72^ Presumably, stimuli such as intestinal inflammation appear to enhance transintestinal cholesterol reflux.^73^ Additionally, there is evidence that chronic inflammation changes LDL subclass levels. Patients with chronic inflammatory diseases such as psoriasis, rheumatoid arthritis, ankylosing spondylitis, and IBD had significantly higher levels of small dense LDL.^74^ Remarkably, neither anti-tumor necrosis factor nor anti-interleukin-6 receptor therapy affected levels of small dense LDL, showing that pathways other than inflammation may also be involved.^75^ Koutroumpakis et al^16^ described in a cohort of 701 patients with IBD that low total cholesterol and high TG levels are more frequent in IBD (in particular CD) compared with healthy control individuals and are independently associated with more severe disease. High hepatic TG secretion might contribute to smaller LDL particles and increased VLDL TG, as observed in our CD patients (Figure 3). Although the atherogenic effect of LDL on small and large peripheral vessels and coronary arteries has been well studied,^76^ a differentiated LDL subclass analysis has rarely been performed. To the best of our knowledge, no LDL subclass determination has been performed in IBD patients to date.

We postulate that a predominance of LDL subclasses with increased density and smaller overall size (LDL-2 to LDL-6) (Figure 2) may favor the increased cardiovascular risk in the IBD study cohort. Determination of LDL subclasses could sharpen the cardiovascular risk profile of patients in this cohort. Data from Duan et al^77^ support this hypothesis by documenting increased levels of LDL-3, LDL-4, and LDL-5 serum concentrations in a cohort with acute ischemic stroke. Moreover, Pan et al^78^ reported that elevated LDL-1 serum concentrations are associated with stable carotid plaques, whereas elevated LDL-3 concentrations are associated with unstable carotid plaques. Kayran et al^79^ also described that LDL-2, LDL-3, and LDL-4 serum concentrations are independent risk factors for developing acute ischemic stroke.

Additionally, ApoA1 and ApoA2 are thought to have vasoprotective properties.^80^ APOA1-mimetic peptides 4F and Tg6F inhibited intestinal inflammation in mouse models. In particular, ApoI blocked proinflammatory effects of lipid polysaccharide–activated macrophages on the intestinal epithelium while inhibiting its proinflammatory effects and removing proinflammatory lipids from the wall.^81^ Furthermore, downregulation of ApoA1 was found in ileal biopsies in CD compared with UC.^82^ Concordantly, ApoA1-mimetic peptides inhibited proinflammatory pathways and reduced inflammation in the gut.^80^ Accordingly, a low ApoA1 level is associated with an elevated fecal calprotectin in our cohort, while low ApoA2 levels are associated with high GSRS scores (Figure 4). Notably, the elevated fecal calprotectin levels and the high GSRS scores in our cohort represent an IBD group with marked disease activity. Thus, whether ApoA1 and/or ApoA2 analysis is a suitable biomarker for intestinal inflammation needs to be further investigated in prospective studies.

There is compelling evidence that inflammation affects lipid metabolism. Given the central role of LDL in lipid metabolism, inflammation, and atherosclerotic disease, strategies to normalize distribution and composition of LDL particles might improve the prognosis of IBD patients. We demonstrated that several Apo, lipoproteins, and amino acids were significantly altered in the serum of IBD patients, particularly in CD (Figures 1 and 2).

Taken together our results are in good agreement with other studies and demonstrate the potential of metabolomics analysis in IBD patients to identify and validate potential biomarkers. Nevertheless, our analyses also showed discrepancies with other studies. Possible influences explaining the different study results are new drug therapies and further cohort-specific effects as disease activity–specific, matrix-specific, and (pre)analysis-specific effects. Further, the discrepancies from the results of this study might potentially be attributed to the less rigorously controlled conditions of the serum sampling. A limitation of our study cohort is that we cannot ensure that all samples of the HC individuals were collected under fasting conditions. Therefore, the metabolite profile could be influenced by external factors such as diet and activity. However, we contend that, based on the cohort’s considerable size, meticulous phenotyping, and utilization of matched control samples, we have achieved insightful findings. These results, despite the acknowledged restrictions, highlight potential correlations and biomarkers for further exploration.

In the future, the metabolomic signature of IBD patients will be an important element to predict disease progression and to make therapeutic decisions with regard to personalized therapy tailored to the disease phenotype.^45,47^

## Conclusions

Serum metabolomes of patients with IBD significantly differ from those of healthy individuals. Metabolomic profiling revealed a shift from large to small LDL particles in patients with IBD. In addition, ApoA1 and ApoA2 were low in serum of patients with higher disease activity. Abnormal lipoprotein composition is thought to play a role in the pathology of IBD and concomitant diseases such as atherosclerosis. Defining a specific profile of metabolites might improve the identification of patients with a particularly high risk for vascular complications who might even benefit from statin therapy. In the future, the goal should be to use NMR analyses to better predict disease progression and monitor response to drug therapy.

## Supplementary data

Supplementary data is available at *Inflammatory Bowel Diseases* online.

izad298_suppl_Supplementary_Tables

## Data Availability

The data underlying this article are available in the article and in its online supplementary material. In addition, the raw metabolite dataset used in this study is available after approval of an application to the corresponding author.
