# Supplementary material for: Unique Metabolomic and Lipidomic Profile in Serum From Patients With Crohn’s Disease and Ulcerative Colitis Compared With Healthy Control Individuals
Source: Inflamm Bowel Dis. 2023 Dec 29;30(12):2405–17. doi: 10.1093/ibd/izad298 (PMC11630276; doi:10.1093/ibd/izad298)
Supplement: izad298_suppl_Supplementary_Tables [file izad298_suppl_supplementary_tables.docx]

**Unique metabolomic and lipidomic profile in serum from patients with Crohn's disease and ulcerative colitis compared to healthy controls**

H.C. Tews a ^†^ (Dr. med. Hauke Christian Tews), F. Schmelter b ^†^ (Dr. rer. nat. Franziska Schmelter), A. Kandulski a (PD Dr. med. Arne Kandulski), C. Buechler a (Prof. Dr. rer. nat. Christa Büchler), S. Schmid a (Dr. med. Stephan Schmid), S. Schlosser a (Dr. med. Sophie Schlosser), T. Elger a (Tanja Elger), J. Loibl a (Johanna Loibl), S. Sommersberger a (Stefanie Sommersberger), T. Fererberger a (Tanja Fererberger), S. Gunawan a (Stefan Gunawan), C. Kunst a (PD Dr. rer. nat. Claudia Kunst), K. Gülow a (PD. Dr. rer. nat. Karsten Gülow), D. Bettenworth d,e (Prof. Dr. med. Dominik Bettenworth), B. Föh c (Dr. rer. nat. Bandik Föh), C. Maaß c, P. Solbach c (PD Dr. med. Philipp Solbach), U.L. Günther f (Prof. Dr. rer. nat. Ulrich L. Günther), S. Derer b (Prof. Dr. rer. nat. Stefanie Derer), J.U. Marquardt c^‡^ (Prof. Dr. med. Jens Uwe Marquardt), C. Sina b, c, g^‡^(Prof. Dr. med. Christian Sina) and M. Müller a^‡^ (Prof. Dr. med. Martina Müller-Schilling)

^a^ Department of Internal Medicine I, Gastroenterology, Hepatology, Endocrinology, Rheumatology and Infectious diseases, University Hospital, Regensburg, Germany

^b^ Institute of Nutritional Medicine, University Medical Center Schleswig-Holstein, Campus Lübeck, Lübeck, Germany

^c^ Department of Medicine I, University Medical Center Schleswig-Holstein, Campus Lübeck, Lübeck, Germany

^d^ University Hospital Münster, Department of Medicine B - Gastroenterology and Hepatology, Münster, Germany

^e^ Practice for internal medicine, Münster, Germany

^f^ Institute of Chemistry and Metabolomics, University of Lübeck, Lübeck, Germany

^g^ Fraunhofer Research Institution for Individualized and Cell-Based Medical Engineering (IMTE), Lübeck, Germany

^†^These authors have contributed equally to this work and share first authorship

^‡^These authors have contributed equally to this work and share senior authorship

**Corresponding author:**

Dr. med. Hauke Christian Tews

Department of Internal Medicine I, Gastroenterology, Hepatology, Endocrinology, Rheumatology and Infectious diseases,

University Hospital, Regensburg,

Franz-Josef-Strauß-Allee 11

93053 Regensburg

Germany

**Phone:** +49 941 944 17164

**Fax:** +49 941 944 7107

**Email:** [Hauke.Tews@ukr.de](mailto:Hauke.Tews@ukr.de)

**Supplementary Material:**

**Table S1: Metadata with sex, age and BMI of the studied cohort.** CD, Crohn´s disease; UC, ulcerative colitis; HC, Healthy control.

|  | n | sex | | age | BMI |
| --- | --- | --- | --- | --- | --- |
|  |  | ♀ | ♂ | [years, mean value + SD] | [kg/m^2^] |
| CD | 55 | 31 (56%) | 24 (44%) | 41.7 ± 13.9 | 24.8 ± 4.6 |
| UC | 34 | 14 (41%) | 20 (59%) | 40.9 ± 14.6 | 24.2 ± 5.8 |
| HC | 40 | 18 (45%) | 22 (55%) | 41.4 ± 14.9 | 24.7 ± 3.0 |
| p-value | CD *vs.* HC  UC *vs.* HC  CD *vs.* UC | | | 0.9254  0.8867  0.8035 | 0.9257  0.6494  0.6274 |

**Table S2:** Metabolites and lipoproteins quantified with Bruker´s IVDr software and their p-values (multiple, unpaired t-test with correction for multiple testing with false discovery rate approach) for the comparison of Crohn´s disease (CD) and Ulcerative colitis patients (UC) with healthy controls (HC) as well as CD *vs.* UC patients. Further p-values for the simple linear regression of patients with low (<150 µg/g) and high (>150 µg/g) calprotectin levels and of IVDr data with GSRS. [VLDL = very low-density lipoprotein, LDL = low density lipoprotein, LDL = intermediate density lipoprotein, HDL = high density lipoprotein, TG = triglyceride, Chol = cholesterol, FC = free cholesterol, PL = phospholipid, apo = apolipoprotein].

|  | **CD *vs.* HC** | **UC *vs.* HC** | **CD *vs.* UC** | **Calprotectin** | **GSRS** |
| --- | --- | --- | --- | --- | --- |
|  | P value (FDR=1%) | P value (FDR=1%) | P value (FDR=5%) | P value (FDR=1%) | P value |
| Ethanol | 0.3966 | 0.2511 | 0.8965 | 0.4741 | 0.4806 |
| Trimethylamine-N-oxide | 0.7573 | 0.3934 | 0.2646 | 0.2507 | 0.2361 |
| 2-Aminobutyric acid | 0.0001 | 0.0128 | 0.1772 | 0.1679 | 0.0407 |
| Alanine | 0.0001 | 0.0013 | 0.9202 | 0.2441 | 0.5017 |
| Asparagine | 0.0559 | 0.1389 | 0.8201 | 0.1542 | 0.4935 |
| Creatine | 0.0727 | 0.6920 | 0.3015 | 0.6256 | 0.4471 |
| Creatinine | 0.3586 | 0.1971 | 0.6889 | 0.1287 | 0.5096 |
| Glutamic acid | <0.000001 | 0.0055 | 0.3437 | 0.0089 | 0.2789 |
| Glutamine | 0.0002 | 0.0090 | 0.6037 | 0.0940 | 0.8564 |
| Glycine | 0.3187 | 0.0168 | 0.3423 | 0.8821 | 0.3527 |
| Histidine | 0.0000 | 0.0001 | 0.7897 | 0.1011 | 0.6483 |
| Isoleucine | 0.0192 | 0.0176 | 0.6897 | 0.1306 | 0.1204 |
| Leucine | 0.0000 | 0.0001 | 0.5809 | 0.3209 | 0.2296 |
| Lysine | 0.0595 | 0.0034 | 0.0831 | 0.5764 | 0.8171 |
| Methionine | <0.000001 | <0.000001 | 0.2526 | 0.3792 | 0.5318 |
| N,N-Dimethylglycine | 0.1362 | 0.0171 | 0.2113 | 0.1408 | 0.7554 |
| Ornithine | <0.000001 | 0.0009 | 0.1379 | 0.6218 | 0.7478 |
| Phenylalanine | 0.0004 | 0.0224 | 0.7375 | 0.0266 | 0.2624 |
| Proline | 0.0020 | 0.0215 | 0.5523 | 0.1032 | 0.1235 |
| Sarcosine | 0.3866 | 0.7378 | 0.5340 | 0.2065 | 0.3434 |
| Threonine | 0.0004 | 0.2180 | 0.0766 | 0.0914 | 0.3999 |
| Tyrosine | 0.0010 | 0.0513 | 0.4814 | 0.1691 | 0.7233 |
| Valine | 0.0000 | 0.0001 | 0.8759 | 0.0399 | 0.0114 |
| 2-Hydroxybutyric acid | 0.2276 | 0.1043 | 0.5513 | 0.3074 | 0.1575 |
| Acetic acid | 0.2409 | 0.8635 | 0.3403 | 0.2101 | 0.5896 |
| Citric acid | 0.0001 | 0.0651 | 0.0624 | 0.7495 | 0.8264 |
| Formic acid | 0.1801 | 0.9084 | 0.3067 | 0.0307 | 0.1495 |
| Lactic acid | 0.0000 | 0.0034 | 0.2684 | 0.0022 | 0.6037 |
| Succinic acid | 0.0944 | 0.0460 | 0.4087 | 0.6316 | 0.4326 |
| Choline |  | 0.1003 | 0.0535 | 0.4741 | 0.9769 |
| 2-Oxoglutaric acid |  | 0.1021 | 0.0548 |  | 0.2979 |
| 3-Hydroxybutyric acid | 0.1921 | 0.2399 | 0.8538 | 0.9698 | 0.7304 |
| Acetoacetic acid | 0.0970 | 0.0224 | 0.8876 | 0.6822 | 0.9124 |
| Acetone | 0.1844 | 0.2606 | 0.8264 | 0.4642 | 0.9930 |
| Pyruvic acid | 0.0000 | <0.000001 | 0.5275 | 0.1069 | 0.5264 |
| D-Galactose | 0.2430 | 0.3904 |  |  |  |
| Glucose | 0.0005 | 0.0092 | 0.8175 | 0.1551 | 0.4459 |
| Glycerol | 0.0003 | 0.0118 | 0.7442 | 0.1555 | 0.3444 |
| Dimethylsulfone | 0.3511 | 0.2491 | 0.9709 | 0.2680 | 0.6792 |
| TG | 0.0481 | 0.5649 | 0.0202 | 0.4552 | 0.3872 |
| Chol | 0.1873 | 0.3823 | 0.8454 | 0.0001 | 0.1080 |
| ApoA1 | 0.5697 | 0.2782 | 0.1187 | 0.0000 | 0.5002 |
| ApoA2 | 0.5740 | 0.1026 | 0.0383 | 0.0000 | 0.0068 |
| ApoB100 | 0.7284 | 0.1723 | 0.2415 | 0.0874 | 0.0781 |
| LDL/HDL | 0.1490 | 0.2367 | 0.0629 | 0.2809 | 0.1458 |
| ApoB100/ApoA1 | 0.9166 | 0.1518 | 0.1414 | 0.3890 | 0.7452 |
| ApoB Particles | 0.7282 | 0.1723 | 0.2415 | 0.0874 | 0.0781 |
| VLDL Particles | 0.0024 | 0.2743 | 0.1483 | 0.9534 | 0.4024 |
| IDL Particles | 0.2578 | 0.3007 | 0.5521 | 0.1528 | 0.0868 |
| LDL Particles | 0.6410 | 0.0935 | 0.0375 | 0.1080 | 0.1125 |
| LDL-1 Particles | 0.0089 | 0.1781 | 0.0071 | 0.4488 | 0.3353 |
| LDL-2 Particles | 0.0000 | 0.5763 | 0.0005 | 0.4844 | 0.8943 |
| LDL-3 Particles | 0.0000 | 0.6773 | 0.0013 | 0.0664 | 0.3373 |
| LDL-4 Particles | 0.0466 | 0.9054 | 0.1671 | 0.0112 | 0.0386 |
| LDL-5 Particles | 0.0467 | 0.1977 | 0.7464 | 0.1053 | 0.1095 |
| LDL-6 Particles | 0.0002 | 0.0044 | 0.5952 | 0.6035 | 0.4486 |
| VLDL TG | 0.0161 | 0.5879 | 0.0085 | 0.4022 | 0.3862 |
| IDL TG | 0.0263 | 0.4981 | 0.0124 | 0.3897 | 0.4242 |
| LDL TG | 0.0328 | 0.0238 | 0.0895 | 0.2218 | 0.4138 |
| HDL TG | 0.0010 | 0.3442 | 0.0310 | 0.4632 | 0.5501 |
| VLDL | 0.0614 | 0.5997 | 0.0184 | 0.2594 | 0.3522 |
| IDL | 0.4023 | 0.7841 | 0.7475 | 0.1040 | 0.0717 |
| LDL | 0.0551 | 0.7357 | 0.1989 | 0.0028 | 0.1322 |
| HDL | 0.6846 | 0.3914 | 0.5595 | 0.0028 | 0.9172 |
| VLDL FC | 0.0069 | 0.6840 | 0.0044 | 0.2378 | 0.4185 |
| IDL FC | 0.4938 | 0.8913 | 0.7268 | 0.0678 | 0.0516 |
| LDL FC | 0.0047 | 0.9935 | 0.0133 | 0.0120 | 0.2283 |
| HDL FC | 0.0176 | 0.0821 | 0.8402 | 0.0043 | 0.5939 |
| VLDL PL | 0.0005 | 0.9009 | 0.0021 | 0.2794 | 0.4778 |
| IDL PL | 0.1234 | 0.4703 | 0.0458 | 0.0220 | 0.1209 |
| LDL PL | 0.1667 | 0.5118 | 0.0674 | 0.0063 | 0.1137 |
| HDL PL | 0.3068 | 0.7181 | 0.2139 | 0.0019 | 0.9866 |
| HDL ApoA1 | 0.4221 | 0.3861 | 0.1269 | 0.0000 | 0.5306 |
| HDL ApoA2 | 0.4295 | 0.2520 | 0.0806 | 0.0000 | 0.0051 |
| VLDL ApoB | 0.0024 | 0.2747 | 0.1481 | 0.9527 | 0.4023 |
| IDL ApoB | 0.2578 | 0.3006 | 0.5520 | 0.1524 | 0.0869 |
| LDL ApoB | 0.6412 | 0.0935 | 0.0375 | 0.1080 | 0.1125 |
| VLDL-1 TG | 0.1494 | 0.1053 | 0.0077 | 0.4437 | 0.3353 |
| VLDL-2 TG | 0.0024 | 0.5746 | 0.0195 | 0.4956 | 0.6025 |
| VLDL-3 TG | 0.0008 | 0.2952 | 0.0213 | 0.5681 | 0.7755 |
| VLDL-4 TG | 0.0000 | 0.0583 | 0.0070 | 0.6218 | 0.9351 |
| VLDL-5 TG | 0.0054 | 0.3400 | 0.0010 | 0.9815 | 0.4611 |
| VLDL-1 Chol | 0.5976 | 0.0466 | 0.0106 | 0.2737 | 0.2732 |
| VLDL-2 Chol | 0.0563 | 0.5948 | 0.1833 | 0.4082 | 0.3608 |
| VLDL-3 Chol | 0.0061 | 0.5147 | 0.0512 | 0.2784 | 0.4468 |
| VLDL-4 Chol | 0.0002 | 0.0490 | 0.2059 | 0.3418 | 0.4647 |
| VLDL-5 Chol | 0.0028 | 0.7297 | 0.0030 | 0.6927 | 0.5954 |
| VLDL-1 FC | 0.1347 | 0.1961 | 0.0101 | 0.1473 | 0.1616 |
| VLDL-2 FC | 0.2718 | 0.9944 | 0.2700 | 0.6416 | 0.3698 |
| VLDL-3 FC | 0.0208 | 0.9052 | 0.0333 | 0.5960 | 0.4803 |
| VLDL-4 FC | 0.3058 | 0.9690 | 0.4731 | 0.1643 | 0.3046 |
| VLDL-5 FC | 0.0144 | 0.1076 | 0.0004 | 0.9395 | 0.8995 |
| VLDL-1 PL | 0.0968 | 0.1907 | 0.0092 | 0.3936 | 0.3334 |
| VLDL-2 PL | 0.0013 | 0.4408 | 0.0232 | 0.4420 | 0.5263 |
| VLDL-3 PL | 0.0006 | 0.4616 | 0.0138 | 0.3677 | 0.5473 |
| VLDL-4 PL | 0.0001 | 0.1246 | 0.0433 | 0.4122 | 0.6147 |
| VLDL-5 PL | 0.0024 | 0.1912 | 0.0002 | 0.7905 | 0.7503 |
| LDL-1 TG | 0.1592 | 0.0750 | 0.1394 | 0.1695 | 0.4202 |
| LDL-2 TG | 0.2372 | 0.1000 | 0.0243 | 0.1206 | 0.6000 |
| LDL-3 TG | 0.1032 | 0.0059 | 0.0147 | 0.2365 | 0.6756 |
| LDL-4 TG | 0.2534 | 0.0557 | 0.0632 | 0.3066 | 0.3307 |
| LDL-5 TG | 0.0062 | 0.0055 | 0.2229 | 0.6918 | 0.2854 |
| LDL-6 TG | 0.0000 | 0.0001 | 0.8626 | 0.2582 | 0.9012 |
| LDL-1 Chol | 0.0002 | 0.7871 | 0.0083 | 0.0149 | 0.2160 |
| LDL-2 Chol | <0.000001 | 0.1222 | 0.0056 | 0.1824 | 0.6208 |
| LDL-3 Chol | <0.000001 | 0.0712 | 0.0083 | 0.0086 | 0.3615 |
| LDL-4 Chol | 0.0303 | 0.4257 | 0.2981 | 0.0063 | 0.0923 |
| LDL-5 Chol | 0.1728 | 0.4600 | 0.7190 | 0.0313 | 0.0792 |
| LDL-6 Chol | 0.0002 | 0.0015 | 0.8474 | 0.4982 | 0.4968 |
| LDL-1 FC | 0.0006 | 0.5766 | 0.0028 | 0.0166 | 0.3442 |
| LDL-2 FC | 0.0000 | 0.1257 | 0.0042 | 0.1690 | 0.5834 |
| LDL-3 FC | 0.0000 | 0.2603 | 0.0036 | 0.0350 | 0.5946 |
| LDL-4 FC | 0.0101 | 0.3141 | 0.2966 | 0.0030 | 0.0723 |
| LDL-5 FC | 0.1637 | 0.3631 | 0.8406 | 0.0253 | 0.1095 |
| LDL-6 FC | 0.0002 | 0.0020 | 0.7379 | 0.1974 | 0.3963 |
| LDL-1 PL | 0.0024 | 0.3875 | 0.0120 | 0.0557 | 0.2801 |
| LDL-2 PL | <0.000001 | 0.2528 | 0.0008 | 0.2846 | 0.6648 |
| LDL-3 PL | 0.0000 | 0.2270 | 0.0036 | 0.0203 | 0.3642 |
| LDL-4 PL | 0.0616 | 0.6604 | 0.3010 | 0.0067 | 0.0552 |
| LDL-5 PL | 0.1099 | 0.4356 | 0.6265 | 0.0381 | 0.0903 |
| LDL-6 PL | 0.0001 | 0.0017 | 0.6844 | 0.4989 | 0.5951 |
| LDL-1 ApoB | 0.0088 | 0.1782 | 0.0071 | 0.4494 | 0.3349 |
| LDL-2 ApoB | 0.0000 | 0.5765 | 0.0005 | 0.4846 | 0.8942 |
| LDL-3 ApoB | 0.0000 | 0.6780 | 0.0013 | 0.0665 | 0.3368 |
| LDL-4 ApoB | 0.0466 | 0.9051 | 0.1672 | 0.0112 | 0.0386 |
| LDL-5 ApoB | 0.0466 | 0.1973 | 0.7470 | 0.1053 | 0.1096 |
| LDL-6 ApoB | 0.0002 | 0.0044 | 0.5951 | 0.6036 | 0.4485 |
| HDL-1 TG | 0.0106 | 0.1521 | 0.3234 | 0.8740 | 0.7723 |
| HDL-2 TG | 0.0102 | 0.9946 | 0.0269 | 0.8210 | 0.8997 |
| HDL-3 TG | 0.0010 | 0.9533 | 0.0045 | 0.5198 | 0.3748 |
| HDL-4 TG | 0.0001 | 0.4961 | 0.0043 | 0.1873 | 0.1085 |
| HDL-1 Chol | 0.9450 | 0.4992 | 0.4541 | 0.3637 | 0.1774 |
| HDL-2 Chol | 0.2397 | 0.0338 | 0.2580 | 0.0282 | 0.5345 |
| HDL-3 Chol | 0.5717 | 0.0256 | 0.0418 | 0.0000 | 0.2659 |
| HDL-4 Chol | 0.6771 | 0.5892 | 0.3549 | 0.0003 | 0.0262 |
| HDL-1 FC | 0.0282 | 0.4318 | 0.3546 | 0.0259 | 0.5008 |
| HDL-2 FC | 0.0214 | 0.0234 | 0.7885 | 0.0027 | 0.8523 |
| HDL-3 FC | 0.8181 | 0.3200 | 0.1910 | 0.0000 | 0.0972 |
| HDL-4 FC | 0.1025 | 0.9666 | 0.1754 | 0.0000 | 0.0442 |
| HDL-1 PL | 0.7519 | 0.4475 | 0.5913 | 0.3783 | 0.1962 |
| HDL-2 PL | 0.9180 | 0.2423 | 0.2106 | 0.0716 | 0.6463 |
| HDL-3 PL | 0.1479 | 0.3460 | 0.0178 | 0.0003 | 0.1905 |
| HDL-4 PL | 0.0441 | 0.7960 | 0.0618 | 0.0000 | 0.0203 |
| HDL-1 ApoA1 | 0.9542 | 0.5697 | 0.5312 | 0.3019 | 0.2540 |
| HDL-2 ApoA1 | 0.0857 | 0.9181 | 0.1636 | 0.0097 | 0.8295 |
| HDL-3 ApoA1 | 0.6495 | 0.0398 | 0.0098 | 0.0001 | 0.2267 |
| HDL-4 ApoA1 | 0.1982 | 0.5609 | 0.1102 | 0.0000 | 0.0172 |
| HDL-1 ApoA2 | 0.2559 | 0.3863 | 0.9905 | 0.1968 | 0.4460 |
| HDL-2 ApoA2 | 0.0995 | 0.6423 | 0.0555 | 0.0040 | 0.4731 |
| HDL-3 ApoA2 | 0.6037 | 0.0704 | 0.0285 | 0.0002 | 0.0334 |
| HDL-4 ApoA2 | 0.4536 | 0.4587 | 0.1888 | 0.0004 | 0.0022 |
